# Fabrication Method Study of ZnO Nanocoated Cellulose Film and Its Piezoelectric Property

**DOI:** 10.3390/ma10060611

**Published:** 2017-06-02

**Authors:** Hyun-U Ko, Hyun Chan Kim, Jung Woong Kim, Lindong Zhai, Jaehwan Kim

**Affiliations:** Creative Research Center for Nanocellulose Future Composites, Department of Mechanical Engineering, Inha University, Incheon 22212, South Korea; lostmago@naver.com (H.-U.K.); Kim_HyunChan@naver.com (H.C.K.); jw6294@naver.com (J.W.K.); duicaofei@naver.com (L.Z.)

**Keywords:** cellulose, ZnO nanocoating, hydrothermal synthesis, piezoelectric charge constant, continuous production

## Abstract

Recently, a cellulose-based composite material with a thin ZnO nanolayer—namely, ZnO nanocoated cellulose film (ZONCE)—was fabricated to increase its piezoelectric charge constant. However, the fabrication method has limitations to its application in mass production. In this paper, a hydrothermal synthesis method suitable for the mass production of ZONCE (HZONCE) is proposed. A simple hydrothermal synthesis which includes a hydrothermal reaction is used for the production, and the reaction time is controlled. To improve the piezoelectric charge constant, the hydrothermal reaction is conducted twice. HZONCE fabricated by twice-hydrothermal reaction shows approximately 1.6-times improved piezoelectric charge constant compared to HZONCE fabricated by single hydrothermal reaction. Since the fabricated HZONCE has high transparency, dielectric constant, and piezoelectric constant, the proposed method can be applied for continuous mass production.

## 1. Introduction

Cellulose is one of the most abundant biopolymers in the world—more than 200 billion tons of lignocellulosics are produced every year [1]. Because of its abundance, cellulose has been traditionally used for resources of paper, paper board, and texture [2]. However, during the last decade, its novel property of piezoelectricity has been paid attention. Although the piezoelectricity of wood was discovered in the 1950s, cellulose has been rediscovered as a piezoelectric material after reporting cellulose based electro-active paper (EAPap) [3,4]. Utilizing the piezoelectric property of cellulose, its possibility of application has been investigated in fields such as actuators, temperature sensors, strain sensors, and speakers, etc. [5,6,7]. Since cellulose-based composites with highly functional nanomaterials are flexible and disposable, efforts have been made for the development of cellulose-based multifunctional nanocomposites for biosensors, chemical sensors, and paper transistors [8,9,10,11]. Especially, inorganic oxide nanomaterials such as tin dioxide (SnO_2_) and titanium dioxide (TiO_2_) can be interacted with cellulose due to abundant hydroxyl (OH) groups of cellulose chains [12]. Zinc oxide (ZnO) has been highly spotlighted due to its piezoelectricity and wide band gap (3.37 eV) property, which is utilized for energy harvesters and semiconducting devices [13,14,15]. 

ZnO-coated cellulose and its application has been reported [16,17]. Researchers have normally grown ZnO on fibrous paper or pulp. Recently, cellulose and ZnO hybrid composites have been studied by nanocoating [18], blending [19], or nanorod growing of ZnO [20] on cellulose films and have been applied in energy harvesters, glucose biosensors, and strain sensors [21,22]. Growing ZnO on cellulose films is advantageous comparing with ZnO-grown fibrous paper or pulp in terms of the piezoelectric property, better mechanical property, and optical transparency of cellulose films. In growing ZnO, ZnO nanoparticles are immobilized on the surface of regenerated cellulose film by a hydrothermal synthesis in which the controlled hydrolysis of a Zn(II)-amine complex leads to the formation of ZnO nanoparticles [23]. The uniform and strong formation of ZnO nanoparticles on the surface of cellulose film is essential in ZnO growth, and a two-step process is adopted to achieve the uniform and strong formation of ZnO—seeding and nanorod growth. The ZnO nanorod grown on the cellulose film showed its improved piezoelectric property [19,22]. However, this ZnO nanorod growth process consumes a large amount of chemicals and takes a long time. Furthermore, its transparency is low because of the ZnO nanorod size and its layer thickness. Thus, an efficient and cheap process for ZnO coating on the cellulose film is necessary for the mass production of cellulose ZnO hybrid nanocomposite without significantly sacrificing its properties. An attempt was successfully made by ZnO nanocoating on the cellulose film (ZONCE) with spin-coating [18]. Although their attempt gave rise to a good piezoelectric property, it is still inappropriate for mass production because its size is limited by the spin-coating. We have the intention to develop continuous ZnO coating on cellulose film, possibly reducing the production cost. 

**Figure 1:**
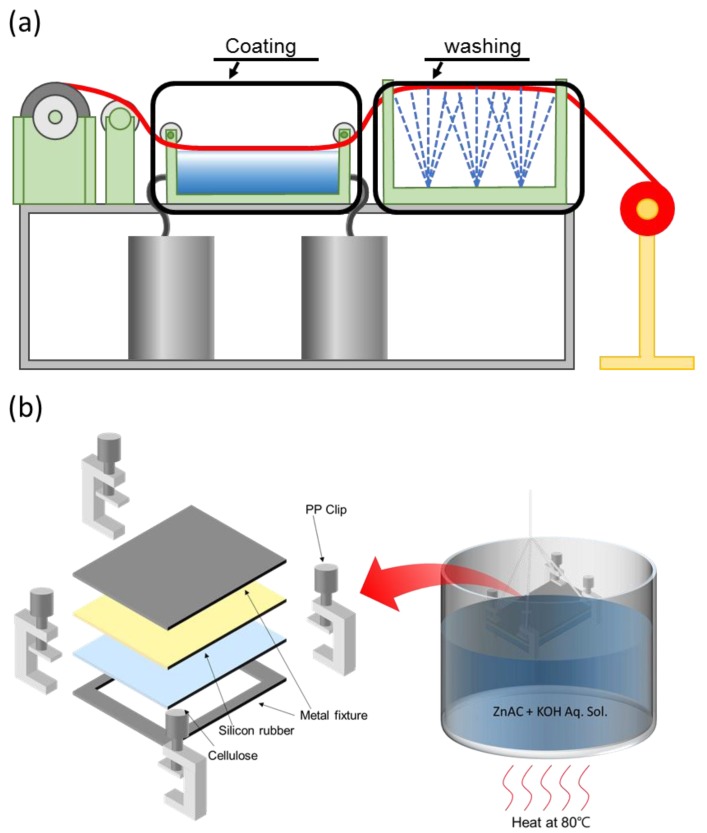
Concepts of (**a**) a continuous production of a hydrothermally synthesized ZnO nanocoating on a cellulose film (HZONCE) and (**b**) laboratory-scale fabrication setup.

Thus, a practical fabrication method for ZnO nanocoated cellulose film (ZONCE) is studied in this paper, which is useful for continuous mass production. Figure 1a shows the concept of continuous mass production of hydrothermally synthesized ZONCE (HZONCE). A wet cellulose film is fed continuously by unwinding. Slowly moving wet film contacts with a ZnO reaction solution in a heating bath. After that, the coated surface is washed with deionized (DI) water, followed by winding and drying. This concept can be implemented by a pilot plant. To prove the concept, a laboratory-scale fabrication setup was made as shown in Figure 1b. Detail of the laboratory setup is explained in the fabrication process. Our aim was to fabricate a thin and uniform ZnO nanocoating layer on the cellulose film less of than 250 nm by the continuous production method without significantly sacrificing the piezoelectric property and optical transparency. A hydrothermal synthesis including thermal hydrolysis reaction is adopted in this research, and the reaction effect is investigated.

## 2. Materials and Methods 

### 2.1. Materials

Cotton pulp with a degree of polymerization (DP) of 4580 was purchased from Buckeye technologies Inc. in Memphis, TN, USA. Lithium chloride (LiCl) with extra pure degree (98.0%) and potassium hydroxide (KOH) were purchased from Deajung Chemicals (Siheung-Si, Gyeonggi-Do, South Korea). *N*,*N*-dimethylacetamide dehydrate (C_4_H_9_NO, DMAc) and Zinc acetate dihydrate (Zn(CH_3_COO)_2_∙2H_2_O, ZnAc) with ACS regent 98% were purchased from Sigma-Aldrich in St. Louis, MO, USA.

### 2.2. Fabrication Process

The wet regenerated cellulose film for HZONCE was prepared by the previously reported method [21]. In brief, cellulose/DMAc/LiCl solution was fabricated by dissolving cotton pulp in DMAc/LiCl solvent system at 150 °C. The cellulose solution was cast on a glass plate and cured in DI water /isopropyl alcohol (IPA) mixture and DI water. As shown in Figure 1b, the wet cellulose film was fixed on a metal fixture to prevent permeation of the ZnO reaction solution on the back side of the cellulose during ZnO coating. The fixture consists of a rectangular stainless steel plate, bezel, silicone rubber, and polypropylene clips.

A ZnO layer was grown on the wet cellulose film by hydrothermal synthesis including thermal hydrolysis reaction. For the process, two different nutrient solutions with precursors were prepared at room temperature. ZnAc was the first precursor for the source of Zn^2+^ ions. For the ZnAc solution, ZnAc was mixed into 100 mL DI water with 50 mM concentration. KOH as source of hydroxide (OH^−^) ions was mixed into 400 mL DI water with 25 mM concentration. The nutrient solutions were mixed to form a reaction solution and heated at 80 °C. The fixed wet cellulose was located to contact with the surface of the ZnO reaction solution. After finishing the reaction, HZONCE was dried in room conditions. Reaction time and number of reactions were varied to investigate resultant piezoelectric properties. In the initial HZONCE fabrication process, the reaction time was varied from 2 to 6 h. In the improved HZONCE fabrication process, the varied reaction time was divided into two steps. 

### 2.3. Characterization

**Figure 2:**
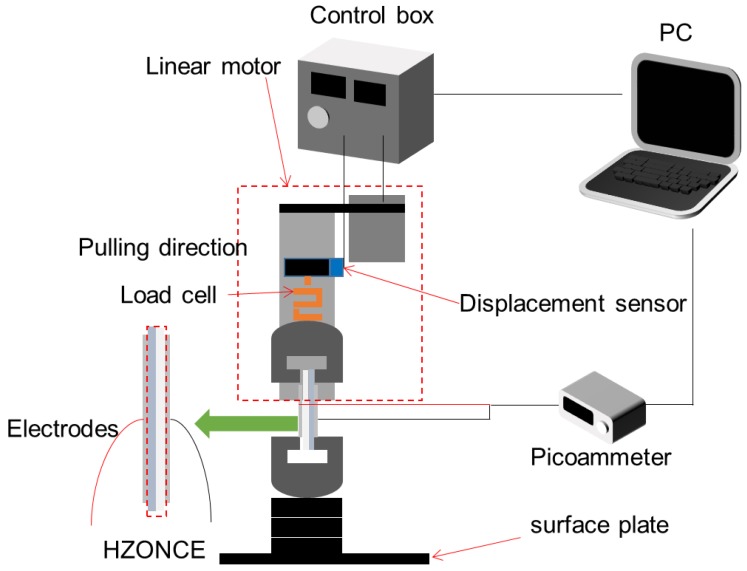
Experimental setup for pull test.

To investigate the morphology of HZONCE, an atomic force microscope (AFM, Dimension-3000, Veeco, Plainview, NY, USA) was used. The transparency of HZONCE was investigated by using a diode array UV visible spectrophotometer (UV-vis, 8452A, HP, Palo Alto, CA, USA). Dielectric property was measured by using an inductance, capacitance and resistance (LCR) meter (4284A, Agilent, Santa Clara, CA, USA). To measure the piezoelectric charge constant and Young’s modulus, the quasi-static pull test was used [24]. Figure 2 shows the pull test system. The pull test system consists of a linear motor (GB-BA/SR128-015, Sony, Minato, Tokyo, Japan) for longitudinal pulling, load cell (UU-K0101, Dacell, Cheangiu-Si, Chungcheong buk-Do, South Korea) to measure pulling force, and picoammeter (6487, Keithley, Solon, OH, USA) to measure induced piezoelectric charge. Samples were prepared to 6 × 1 cm^2^ size. To collect piezoelectric charge, 4 × 1 cm^2^ aluminum electrodes were deposited on both sides of samples. Pulling speed was set to 0.0005 mm/s.

## 3. Results

### 3.1. Initial HZONCE

**Figure 3:**
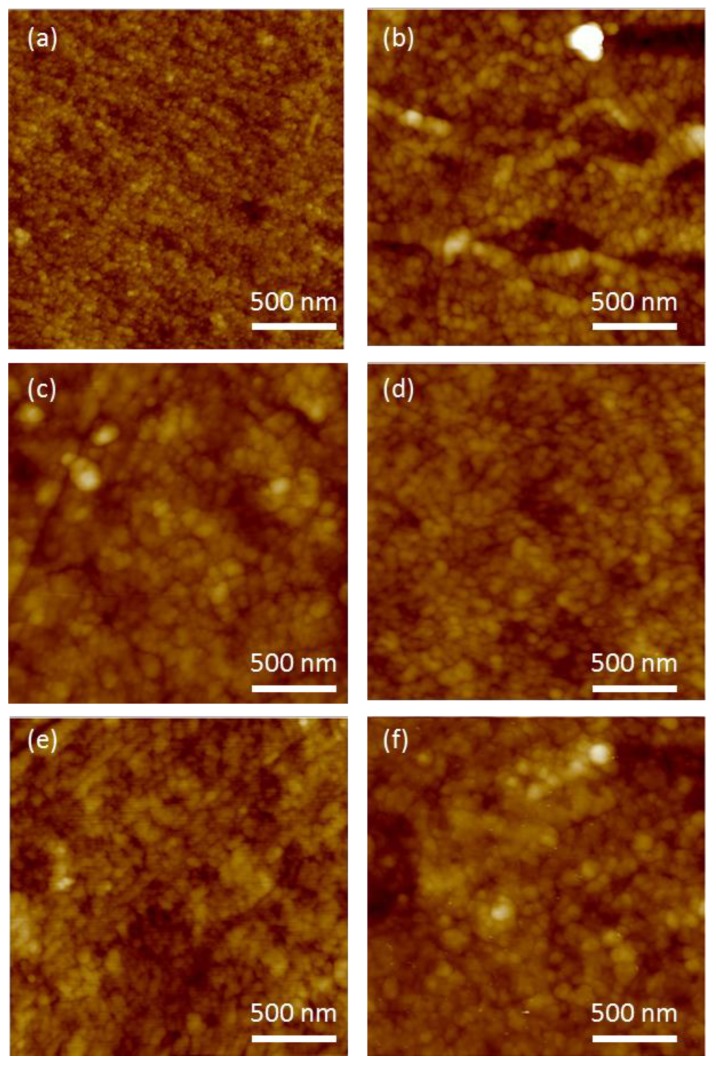
Atomic force microscopy (AFM) images of the initial HZONCEs depending on the reaction time: (**a**) cellulose; (**b**) 2 h; (**c**) 3 h; (**d**) 4 h; (**e**) 5 h; and (**f**) 6 h.

HZONCE was prepared with various reaction times from 2 to 6 h. Figure 3 shows AFM images of the cellulose film and HZONCEs depending on the reaction time. The cellulose film showed quite a uniform surface (Figure 3a). AFM images of HZONCEs show nanosized ZnO grains (about 50 nm), and its density increased as the reaction time increased. The existence of a ZnO nanolayer cannot be seen by scanning electron microscope, and only AFM or transmission electron microscopes can see it. 

**Figure 4:**
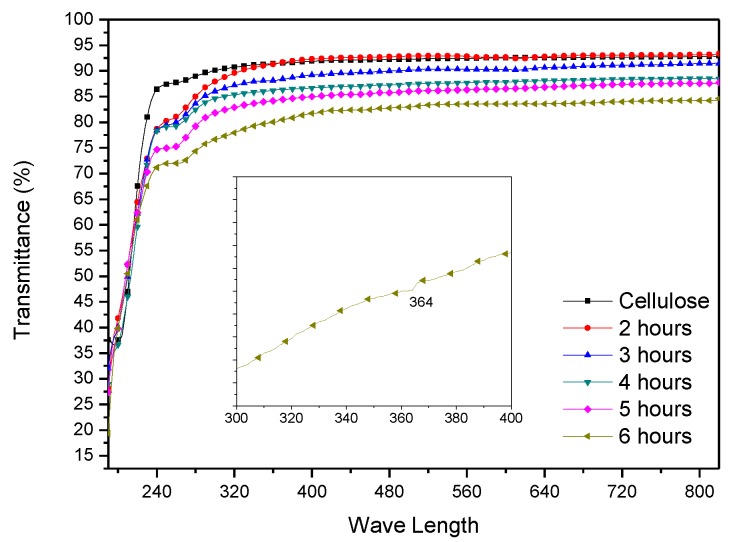
Transparency of the initial HZONCEs depending on the reaction time.

Figure 4 shows UV-vis transmission spectra taken by UV-visible spectrometer. Transmittance of the cellulose film was around 90% in the visible range. The UV-visible spectrum of 6 h HZONCE shows an absorption peak at 364 nm wavelength, which confirms the ZnO nanolayer deposition on the cellulose surface [25]. As the reaction time increased, the transmittance of HZONCE decreased, but not below 80% in the visible range. This indicates that the amount of ZnO nanoparticles grown on the surface of the cellulose film increased as the reaction time increased. 

**Figure 5:**
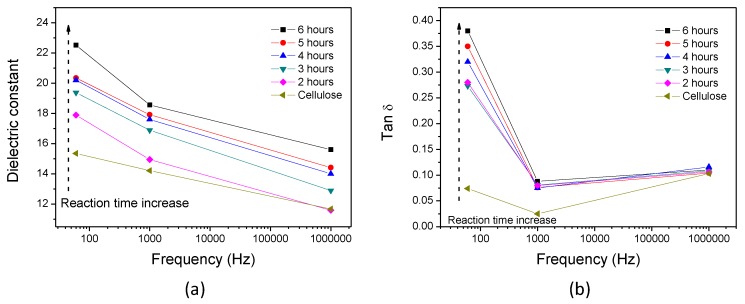
(**a**) Dielectric constant and (**b**) dielectric tan δ of the initial HZONCEs depending on the reaction time.

Figure 5 illustrates dielectric constant and dielectric dissipation factor depending on the reaction time at 60 Hz, 1 kHz, and 1 MHz. Dielectric constant was measured at room temperature conditions. Figure 5a shows the dielectric constants of HZONCEs and the cellulose film. Dielectric constant increased from 17.9 to 22.5 as the reaction time changed from 2 to 6 h at 60 Hz. Note that the dielectric constant of the cellulose film was 15.3 at 60 Hz. The dielectric constant result indicates that ZnO nanolayer improved the polarity of cellulose. Figure 5b shows the dielectric dissipation factors of cellulose and HZONCEs. The dielectric dissipation factor of HZONCE increased from 0.28 to 0.38 as the reaction time increased from 2 to 6 h at 60 Hz, which is larger than the cellulose film (0.07). This result indicates that the interfacial polarity increases between the cellulose film and ZnO nanoparticles. Dielectric dissipation factors of HZONCEs are shown to be 0.08−0.09 at 1 kHz. The reduction of the dielectric dissipation factors implies the reduction of the interfacial polarity effect on the polarity of materials. Dielectric dissipation factors of cellulose and HZONCEs at 1 MHz increased again to 0.11, which means that interfacial polarization effect was eliminated at 1 MHz. Although the effect of interfacial polarity was eliminated, the dielectric constants of HZONCEs were higher than that of the cellulose film in the entire frequency range. Regarding the reaction time effect, the dielectric constants of HZONCEs increased with the reaction time. It is clear that as the reaction time increased, the ZnO nanolayer thickness increased, resulting in dielectric constant enhancement. The reason why the dielectric property of HZONCE is lower than that of ZNOCE might be mainly due to the thickness of the ZnO nanolayer. According to a previous report [18], the improvement of the piezoelectric charge constant of ZONCE was associated with three reasons: the dipolar orientation associated with the crystal structures of ZnO and cellulose; the size effects in ZnO nanoseeds; and flexoelectricity of the ZnO nanolayer associated with strain due to shrinkage of the cellulose film. After drying, the cellulose film shrinks a little, but the ZnO layer does not. This results in a slight bend in ZONCE. This bending introduces so-called flexoelectricity [26,27]. In other words, the ZnO nanolayer is under compression stress when ZONCE is flat. Note that the thickness of the ZnO nanolayer in ZONCE was 70–85 nm and was well deposited on the surface of cellulose. On the other hand, the thickness of the ZnO nanolayer in HZONCE was about 5 nm, and many nanopores were shown (this thickness will be explained in Figure 8). The size effect is associated with charge redistribution near the free surfaces leading to changes in local polarization [28]. The thickness decrease of the ZnO nanolayer means electron donor decrease. Furthermore, the porous behavior of the ZnO layer in HZONCE might be associated with the reduced dielectric and piezoelectric properties. Thus, the dielectric and piezoelectric properties of ZONCE at low frequency are higher than those of the improved HZONCE. Although the dielectric and piezoelectric properties of the improved HZONCE are less than those of ZONCE, the proposed fabrication method for the improved HZONCE is worthy of mass production.

**Figure 6:**
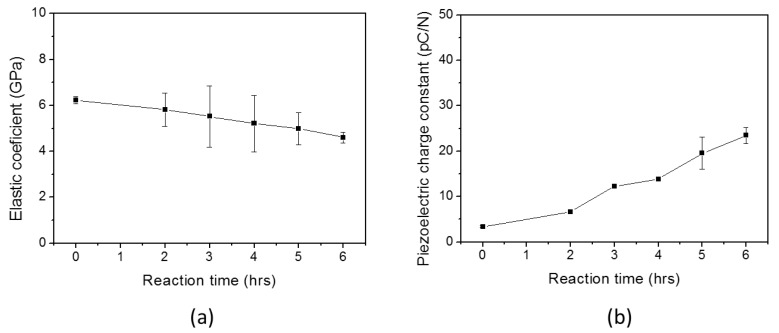
(**a**) Young’s modulus and (**b**) piezoelectric charge constant of the initial HZONCEs depending on the reaction time.

The Young’s moduli of cellulose and HZONCEs were analyzed by using the pull test system. Figure 6a shows the Young’s modulus of HZONCEs depending on the reaction time. “0 h” indicates the cellulose film without ZnO nanocoating. The Young’s modulus slowly decreased from 5.82 to 4.60 GPa as the reaction time increased from 2 h to 6 h. This might be due to stress concentration between the different elastic properties of the ZnO layer and the cellulose film. 

To study the piezoelectric property, a quasi-static test was conducted by using the pull test system. Figure 6b shows piezoelectric charge constant of HZONCEs with the reaction time. The piezoelectric charge constants of cellulose and HZONCEs increased from 3.40 to 23.44 pC/N as the reaction time increased from 2 h to 6 h. The 6 h-reacted sample showed a piezoelectric charge constant seven times higher than the cellulose film (0 h). Note that the piezoelectric charge constant of the cellulose film is very low because it was not mechanically stretched. The piezoelectric charge constant of the 6 h-reacted HZONCE is similar to the mechanically stretched cellulose [18]. This result is due to increased dipole charge. Normally, increasing the dielectric constant leads to an improvement of the piezoelectric charge constant. In summary, dielectric, electromechanical, and optical properties of HZONCE were compared with those of ZONCE [18] as shown in Table 1. Young’s modulus was rather increased, but dielectric constant and optical transparency of HZONCE were a bit lower than those of ZNONCE. The piezoelectric charge constant of HZONCE was shown to be lower than that of ZONCE. 

### 3.2. Improved HZONCE

**Figure 7:**
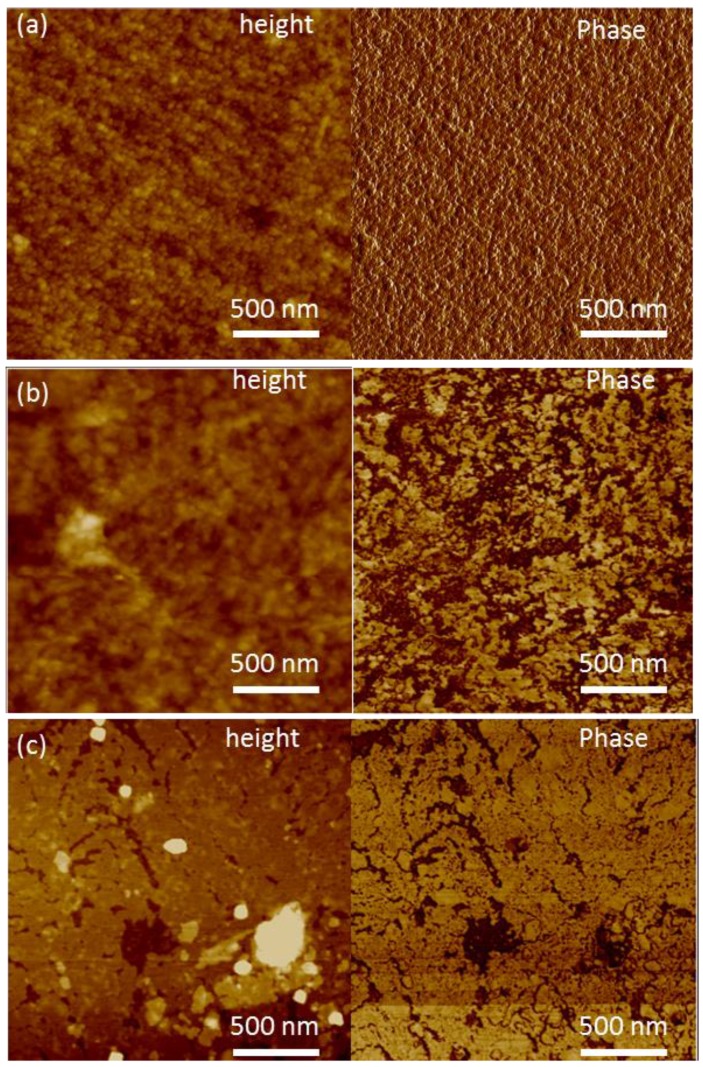
AFM images in terms of height and phase for the improved HZONCEs depending on the reaction time: (**a**) cellulose; (**b**) twice-1 h; (**c**) twice-3 h.

**Figure 8:**
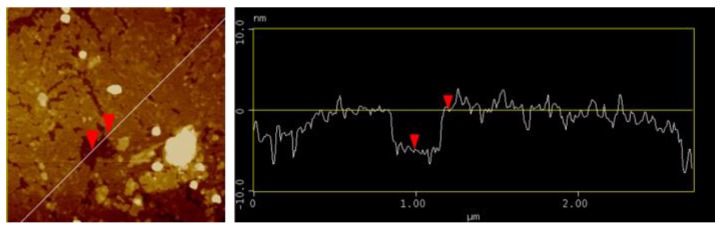
A line profile for twice-3 h case of the improved HZONCE.

To improve the properties of HZONCE, twice-hydrothermal synthesis was conducted. Twice-hydrothermal synthesis means that the hydrothermal synthesis time was divided into two steps and the reaction solution was entirely replaced with new reaction solution for the second step. Reaction time was varied as twice of 1 h, 2 h, and 3 h, which are the same as 2 h, 4 h, and 6 h of the initial HZONCE process. Figure 7 shows AFM images in terms of amplitude and phase of the cellulose film and the improved HZONCEs with different reaction times. In the height image of the twice-1 hour reaction case, small ZnO nanoparticles were observed. However, its phase image is different from the cellulose film. In the phase image of the cellulose, the phase is almost homogeneous on the entire surface of the film. In the twice-1 hour reaction case, it is distinguished by two different phase angles. As the reaction time increased to twice-3 h, ZnO nanoparticle size became small. Note that brighter color indicates large phase change in the AFM phase images. This result shows that the stiff ZnO effect on the mechanical property of HZONCE surface was increased by the increase of reaction time. In summary, ZnO nanoparticle size reduction and the mechanical property improvement of HZONCE were obtained by twice-hydrothermal synthesis. A line profile shown in Figure 8 indicates that the thickness of the ZnO layer is 5 nm. It was estimated that ZnO on the initial HZONCE was dissolved in highly basic reaction solution and re-deposited on ZnO. Because of ZnO dissolving, Zn^2+^ and OH^-^ ions reached near the cellulose surface. So, small-size particles were formed [29]. 

**Figure 9:**
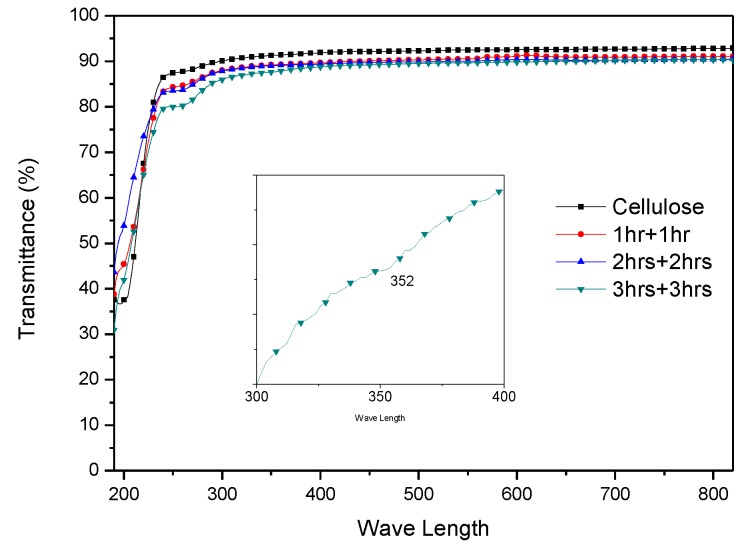
Transparency of the improved HZONCEs depending on the reaction time.

Transparency of the improved HZONCE is almost 90%, which is similar to the cellulose film, as shown in Figure 9. When comparing with the improved HZONCE of twice-3 h and the initial HZONCE of 6 h shown in Figure 6, the transparency of twice-3 h case was higher than the 6 h case. This is due to the smaller size of ZnO. The wavelength of the absorption peak for the improved HZONCE was reduced to 352 nm. This is due to smaller size of ZnO [30]. The UV-vis result confirms ZnO nanocoating on the cellulose surface by hydrothermal reaction. 

**Figure 10:**
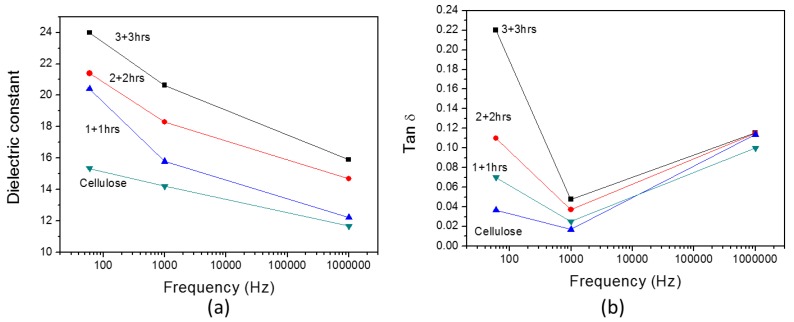
Dielectric properties of the improved HZONCE: (**a**) dielectric constant; and (**b**) dielectric tan δ.

Figure 10 shows the dielectric constant and tan δ of the improved HZONCEs. The dielectric constant of the improved HZONCEs increased from 20.4 to 24.0 as the reaction time increased from twice-1 hour to twice-3 h. These values are higher than those of the initial HZONCEs. The dielectric dissipation factors of the improved HZONCEs showed lower values than the initial HZONCEs. Thus, this result indicates that twice-hydrothermal synthesis improved the dielectric properties of HZONCE.

**Figure 11:**
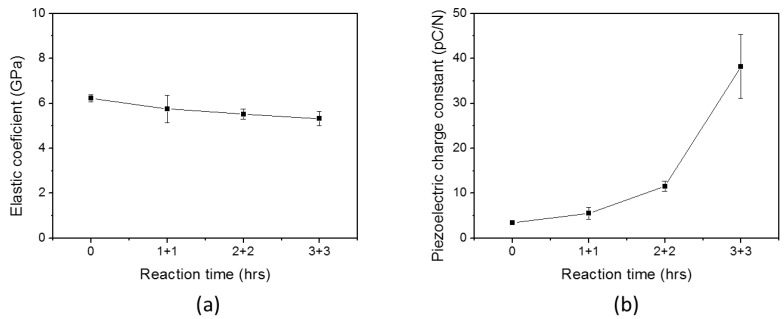
(**a**) Young’s modulus and (**b**) piezoelectric charge constant of twice HZONCEs depending on reaction time.

Figure 11a shows Young’s modulus of the improved HZONCE; its value decreased from 5.8 to 5.3 GPa as the reaction time increased. Note that the Young’s modulus of the twice-3 h case in the improved HZONCE was higher than the 6 h case in the initial HZONCE. Figure 11b shows the piezoelectric charge constant of the improved HZONCE. As the reaction time increased, the piezoelectric charge constant of the improved HZONCE increased. When comparing the improved HZONCE and the initial HZONCE, the piezoelectric charge constant of the twice-1 h and -2 h cases of the improved HZONCE are smaller than the 2 h and 4 h reacted cases of the initial HZONCE. This might be due to the fact that the ZnO nanolayer was not fully covering the cellulose surface. However, the twice-3 h case of the improved HZONCE increased its piezoelectric charge constant to 37.4 pC/N—almost 150% of the 6 h case of the initial HZONCE. This was due to size of ZnO. Commonly, composite materials with small particles show high dielectric constant [31] because of increased interaction area between particles and the base material. In the improved HZONCE case, the gap between cellulose and ZnO nanoparticles could be very small due to the small size of the ZnO nanoparticles and the larger interaction area compared to the initial HZONCE case. Thus, the dielectric constant and piezoelectric constant were improved. Additionally, the transparency was increased due to the decreased band gap, which agrees with the UV absorption peak shift. The dielectric constant of the improved HZONCE was almost 40% that of the ZONCE case [18]. This performance sacrifice may be acceptable for feasibility of mass production. Table 1 shows the comparison with the initial HZONCE for the 6 h case, the improved HZONCE for the twice-3 h case, and ZONCE. We did not try a twice-4 h case. Based upon the observed tendency, the piezoelectric charge constant for the case is expected to be slightly improved. However, increasing the reaction time consumes more chemicals and increases production time, which hinders mass production. 

## 4. Conclusions

To achieve practical mass production, ZnO nanocoated cellulose films were fabricated by using several hydrothermal synthesis methods (HZONCE). Reaction time and number of reactions were varied, and the optical transparency, mechanical property, and piezoelectric property were investigated. In the initial HZONCE fabrication process, the reaction time was varied from 2 to 6 h, and in the improved HZONCE fabrication process, the varied reaction time was divided into two steps. The tendency of dielectric constant, dielectric loss factor, and piezoelectric charge constant might be associated with the interfacial polarization increase as the reaction time increased. The piezoelectric charge constants of cellulose and the initial HZONCE increased from 3.40 to 23.4 pC/N, which is smaller than the original ZONCE value. In the improved HZONCE fabrication process for the twice-3 h case, the ZnO nanocoating covered the large surface of cellulose and the improved piezoelectric charge constant was shown to be 37.4 pC/N. This result shows that ZnO coating on cellulose by hydrothermal process is possible for continuous mass production. 

## Figures and Tables

**Table 1:** Comparison of the initial HZONCE and the improved HZONCE with ZONCE.

Properties	ZONCE [18]	Initial HZONCE	Improved HZONCE
Young’s modulus (GPa)	3.5	4.6	5.3
Dielectric constant (at 1 kHz)	21.3	18.6	24.0
Piezoelectric charge constant (pC/N)	93.5	23.4	37.4
Optical transparency (at 480 nm)	86.0	82.8	90.0
